# Report of a Case of Acute Nephritis in an Infant

**Published:** 1899-06

**Authors:** G. A. Himmelsbach

**Affiliations:** Buffalo, N. Y., Assistant attending physician, Buffalo General Hospital; attending physician, Erie County Hospital.


### BUFFALO MEDICAL JOURNAL.

VOL. XXXVIII. JUNE, 1899. No. 11.

### Original Communications.

### REPORT OF A CASE OF ACUTE NEPHRITIS IN AN INFANT.—PROBABLE CAUSE SACCHARIN.

BY G. A. HIMMELSBACH, M. D., Buffalo, N. Y.,[1]

Assistant attending physician, Buffalo General Hospital; attending physician, Erie County Hospital.

ON THE morning of Tuesday, January 18, 1898, I was called to see baby E., aged 14 months. Upon arrival, I found the child in his crib presenting a picture of anemia, with considerable lassitude. I learned upon questioning the parents that the baby had not been feeling perfectly well for a few days past, that the same morning he had been down stairs, sat in his high chair at the breakfast table and was playful; it was evident to the parents, however, that the child was ailing. I also learned that the little one had been a sufferer from chronic constipation almost since birth, and for the past few days the bowels had been exceptionally sluggish and slow in responding to laxatives. The father, who is a physician, had given the matter attention and had washed out the bowels and administered purgatives. The temperature was 99½°; pulse, 150; respiration, 42. The child was very anemic, but allowance had to be made because his natural color was very pale, his skin and hair fair.

Owing to the acceleration of the respiration, I made a careful examination of his chest, but with a negative result, the lungs were perfectly clear, normal vesicular breathing in all parts; the cardiac sounds were smooth and well-balanced, the abdomen was soft and relaxed, the organs were easily palpated; no tympany. My attention was called to a stool the parents had saved; it was formed, dark green in color, but not of an offensive odor. I concluded my patient was suffering from some form of toxemia, and it was my belief that the torpidity of the bowels was responsible for it. I learned that the child had been absolutely breast-fed until about two months

1. Read before the Buffalo Academy of Medicine, March 14, 1899.

before, when the natural food supply had become insufficient, and a modified preparation of cow's milk was given besides, consisting of pure cow's milk, crystal water, cream and saccharin; the proportion of these various constituents was varied from time to time as the child grew older. I was informed that on the Thursday preceding my call the breast supply was completely cut off, and that since that time it had been fed exclusively on the modified cow's milk, under strict directions. This fact of weaning the child and the substitution of artificial food, strengthened me in my belief that the bowels and the nature of the food were the causes of the illness. The father told me that he had examined the urine ten days before and found it normal.

The food was taken away at once, albumin water and brandy substituted; calomel in small doses and bismuth were administered internally, and the bowels were washed with a high-up enema. At six o'clock of the same day, I was called again and was told that the child was worse. It was agreed then to have assistance and counsel accompanied me. Upon entering the room I was struck with the marked change which had taken place since my visit at noon; the child was now more profoundly anemic, eyes sunken and deep creases about the face, which were not present at noon; it was dull and listless. All this betokened danger. The history, related before, was given in detail to the counsel and he examined the child carefully; he expressed as his opinion that it was suffering from some form of toxemia, but there were so many symptoms of the case that were conflicting and inharmonious, that he asked for further assistance. This was agreed upon and counsel No. 2 was called.

The case was presented to him, and after a thorough examination he gave as his opinion that the child was not dangerously ill. He admitted that the pathology of the case was not clear, and that in his judgment the child's teeth were in a measure responsible for the dull, listless state; he had seen many children as ill for forty-eight hours with no more pathology than difficult dentition; that four molar teeth were all about the same stage of advancement and that he would not hesitate to make an incision over the external cusp of each of the four teeth, if matters grew worse. At half-past eleven that same night I was summoned again, and upon arrival I found the child failing; so, apprehensive and uneasy as to the outcome, I asked for further help. Counsel No. 3 came at 1 a. m. and went over the child, agreeing with two of the others, that it looked like some form of poisoning; he agreed to the therapeutics already adopted, but added a hot mustard bath and coffee as a stimulant. I was called again at half-past six a. m. and found the child failing rapidly; respiration, 45; pulse, 160 and weak; temperature. 99½° F.; in a deep stupor, difficult to arouse. The father reported to me that twice the child had screeched, rolled its eyes and had some muscular twitching about the face, simulating the forerunner of a convulsion, but it did not have a distinct spasm. It was now perfectly clear to me that all efforts to save the child were of little avail. I

asked the mother about the voidance of water, and her reply was in the affirmative and frequently, and she was sure that it was not the water from the bowels—"rectal lavage" having being used several times. The child grew progressively worse and died at ten minutes past eleven a. m., or within twenty-two hours after my first examination. I was nonplussed for a cause of death, but was relieved from this worry upon being told that the father had willingly consented to a post mortem examination. Dr. H. U. Williams very kindly consented to do the work; assisted by Dr. Kerr, he made an autopsy at five p. m. The result of this most carefully performed necropsy was practically negative. All parts of the body, including the brain, were carefully examined, and with the single exception of a suspicious change in the kidneys,[1] not one or a group of pathological changes could be found to account for the infant's death. No one present cared to express an opinion as to the probable cause of death. During the autopsy it was observed that the bladder was somewhat distended, and at my suggestion and request Dr. Williams gave me a specimen of the urine. We obtained it by holding a sterilised test-tube and forced the urine into it by compressing the bladder. In this way we avoided any contamination with the blood from surrounding parts. I took the specimen to my office and made a painstaking analysis, and to my utter amazement, the pathology of this mysterious death was unfolded. The following is the report of my analysis:

Quantity, ℥i.
Color, lemon yellow.
Transparency, clear.
Deposit, whitish, cloudy.
Reaction, sharply acid.
Density. The amount too small to take specific gravity.
Odor, sweetish, inoffensive.
*Albumin plentiful.*
Micro.
- Renal casts, as many as four in one field. (Hyaline. Epithelial. Granular.)
- Red blood cells, few.
- Leucocytes.
- Many small uric acid crystals.
- Many small epithelial cells.

Thus it will be observed from the foregoing report that the cause of death was nephritis.

In the careful study of this case, two perplexing problems confronted us: (1) The unusualness of renal disease in so young an infant; (2) The probable cause of a primary nephritis in a child of this tender age.

Recalling my history it will be remembered that ten days before the child's death, the father had made an analysis of a single sample of urine and found it to be perfectly normal. This was done without

1. Sections made of the kidneys by Dr. Williams showed acute parenchymatous degeneration.

the slightest idea of discovering anything wrong; the child to all appearance was well, but as a careful, painstaking father and physician, he did it as a matter of routine.

In searching for data and information concerning this disease, one is struck by the scarcity of literature upon the subject; this I am forced to believe is due to the comparative infrequency of the disease, or else the too frequent oversight in diagnosis when it is present.

Holt, Diseases of Infancy and Children, 1897, in speaking of primary nephritis in infancy says: "These cases are not common, and the symptoms are so obscure that they are usually overlooked."

In 1887, Archives of Pediatrics, Holt published five cases of his own and collected from literature fourteen others of primary nephritis, under two years of age, and from 1887 to 1897, the same author has observed four additional cases.

*Study of Cases.*—Symptoms: The onset in nearly every instance was abrupt with high fever, sometimes reaching 104° F., but, as a rule, 101° to 103°; dropsy, very exceptional, and when present is slight and seen only toward the close of the disease; duration, eight days to four weeks; average, two and a half weeks. Sometimes vomiting and diarrhea, but these not prominent; rarely did they exist as symptoms of uremia. Anemia, a pronounced symptom in nearly every instance. It was this symptom that enabled the author in nearly every case to make a correct diagnosis. Nervous symptoms usually prominent. In several instances dyspnea, without any pulmonary disease, partly due no doubt to the anemia. In nearly all cases restlessness and muscular twitching, and in three convulsions. Dulness and apathy in all fatal cases, but deep coma never seen. Urine rarely scanty until toward the end of the disease and sometimes not even then. Suppression of urine occurred in but few cases, albumin was frequently absent early in the attack, but invariably present at a late period, although rarely in large amount. Casts found in all cases, chiefly hyaline, granular and epithelial; no blood casts. Usually many pus cells and renal epithelial cells, together with red blood cells.

Of 23 cases, 15 died and 8 recovered. Of Holt's own 9 cases, 8 were fatal, diagnosis being confirmed by autopsy in every case but one. It is difficult to state whether these figures represent the actual mortality of the disease. No doubt there are many mild cases which escape notice altogether; the severe ones, however, are quite uniformily fatal, chiefly on account of the tender age of the patients.

Contrast these words of Holt, who says the disease is *uncommon*, with those of Jacobi, who published a very suggestive paper in the *New York Medical Journal*, January 18, 1896, and says: "Nephritis is a frequent disease of infancy and childhood and by no means very rare in the newborn. What was formerly considered mere albuminuria, or a transient form of it, we have been taught by improved methods of investigation, mainly by the use of the centrifuge, to recognise as nephritis." Jacobi speaking of the etiology of the newborn sums it up under three headings: (1) Congestive; from feeble circulation, congenital heart disease, asphyxia or exposure to low temperature. (2) Obstructive; from physiologic rapid decomposition of the blood of the newly born, the formation of hematoidin, bilirubin, jaundice, the production of methemoglobin by chemic poisons, such as potassium chlorate or by excessive heat. (3) Irritative; from the presence of uric acid or hematoidin infarctions, of purpuric or other interstitial hemorrhages or of microbes and toxins in the numerous eruptive and infectious maladies and in enteritis.

This brings me to the second proposition of my paper, *i. e.*, the probable etiology of the nephritis in my patient.

In the classification of causes Jacobi speaks of a variety of nephritis caused by some irritant, *e. g.*, microbic or toxins from infectious diseases. I wish to add a fourth form—namely, a drug nephritis.

In detailing the history of my case I mentioned the fact that saccharin had been used as a sweetening agent in the food and upon this special point I wish to dwell for a moment.

This substance was discovered by Fahlberg, 1879, and by Prof. Ira Ramsen, of Johns Hopkins University, about the same time. Its chemical name is anhydro-ortho-sulphamin-benzoic acid. "It is a white crystalline powder, soluble in 200 parts of cold water, more freely in hot water, and abundantly in alcohol and ether. It has no chemical relation to sugars and was named saccharin, a word already applied to one of the sugars, solely on account of its intensely sweet taste, which is said to be 280 times that of sugar. One part in 70,000 of water is distinctly tasted." My little patient was always given water to drink, in addition to its breast food, and in order to make it more palatable, the water was sweetened by the addition of the saccharin. Saccharin was begun at about two months of age and administered uninterruptedly until its death at fourteen months. The last two months of its life, this same agent was added to its artificial food as well as its drink. At first it averaged about one one-sixth grain per diem, this amount, however, was increased until at least four, one-sixth grains and even five one-sixth grains were given daily. This feature of the child's food and drink and habit of life, struck me as being so unusual and so unique in my experience, it occurred to me that possibly it had some bearing as to the etiology of the case.

I communicated with Professor Remsen, one who is responsible for the discovery of this drug and through his courtesy, I was placed in possession of all of the literature to date since its discovery. Strange to say, no case or cases has been reported bearing on the toxic effect. Most of the research and experiments have been in the line of its physiological action on all the digestive organs and other secretions and in point of fact, its physiological effects on most of the functions have been observed and studied. Several investigators have experimented upon themselves, but in not a single instance have I found anything bearing directly of the toxicity of saccharin.

So much is written from a purely chemical standpoint. S. Hirschfield in a thesis entitled, Ein Bertrag zur Sasscarinfrage, Erlanger, 1891, makes a study of saccharin, devoting in Pait I. about thirty pages to its chemistry. Part II. of his paper gives the physiological action, but does not refer to its deleterious effect upon the renal cells. He proves that it passes through the body unaltered by the gastric and intestinal secretions and is eliminated in the urine unchanged.

The author experimented on himself for thirty days with pure saccharin and took as much as two grains (?) a day. During this time he noticed oliguria on several occasions, but without any other troublesome symptoms, but he does not know whether the suppression of urine was due to the saccharin or not, as no other observer has reported it.

Capparoni, an Italian, in 1893, reported the result of his investigations on the action of saccharin in the intestine and concludes his paper briefly as follows:

(1) Chemically speaking, saccharin has not any therapeutic value in typhoid fever, except its action on the diarrhea. (2) Did not note any action on the temperature. (3) Saccharin acts especially in disinfecting the intestines. (4) Saccharin is not broken up in the intestine and passes unaltered through the kidneys. (5) Its antiseptic action on pathogenic bacteria is proved by the wonderful results which the author obtained with the drug in cases of chronic urinary trouble. (6) In slight fevers one grain a day for children and two to three grains a day for adults. Given every day thus, for ten days in succession, it did not produce any disturbance nor any alteration in the urine.

Aducco and Mosso, in a paper in 1886, report their experience in the study of the physiological action of saccharin on dogs and frogs, and among other things say : "Saccharin passes into the urine without change and makes the urine very sweet, and urine rendered sweet by saccharin undergoes putrefaction much more slowly than nonsweetened urine." Saccharin does not pass into other secretions, *e. g.*, saliva or milk, and one half hour after ingestion into the stomach, a sweet flavor is detected in the urine.

From these foregoing reports and from others I might add, one fact must be conceded: That saccharin is taken into the alimentary tract, absorbed as saccharin and eliminated from the kidneys as such. My contention, therefore, is that if such is the case, then I have just grounds to assume that a prolonged administration of this chemical produces a harmful and injurious effect upon the renal cells, for upon these cells is thrown the whole burden of elimination. The serious and injurious effects from the protracted ingestion and elimination of alcohol, lead, ergot, arsenic, etc., is too well known to call for more than mere mention, but this fact, I think only helps to strengthen my assumption.

A noteworthy feature of all these experiments is that all were made on the lower animals and adult human beings, no case having been reported as to its effect on an infant. Another important point is, that all investigations have been of rather short duration, varying from a few days to a few weeks, or at longest a few months.

I wish, finally, to offer my theory—namely, that saccharin was responsible for the renal degeneration in my case. It seems to me that the fact that saccharin is eliminated by the kidneys unaltered, would strengthen my theory, that it is all the more harmful to the kidney structure, and supporting this, I have the endorsement of no less an authority than the discoverer himself, who says in a personal letter to me: "Whether the more delicate organism of a young infant is more susceptible to its action than that of an adult, is a question that I cannot answer. I think that it is quite conceivable that it might do harm in such a case. If anyone had asked me whether it is advisable to administer saccharin to infants, I should have expressed a doubt as to the advisability of so doing, simply because I do not think we know enough about the action of this material. It is said to pass through the system unchanged and evidence has been furnished in cases of adults that this is the truth, but that indicates the possibility of its acting as an irritant in some parts of the body, and even a slight irritant might be a very serious matter to a young infant."

A few suggestions commend themselves to your attention: (1) The cause of death was acute parenchymatous nephritis as proved by the analysis of the urine and Dr. Williams's examination of the kidneys. (2) The prolonged administration of saccharin contributed toward, if it was not entirely responsible for, the renal degeneration. (3) That in all cases of infant's diseases, whether the diagnosis is perfectly clear or obscure, the urine should in every instance be examined.

137 West Tupper Street.

